# Supplementary material for: Integrated Multi-Tumor Radio-Genomic Marker of Outcomes in Patients with High Serous Ovarian Carcinoma
Source: Cancers (Basel). 2020 Nov 17;12(11):3403. doi: 10.3390/cancers12113403 (PMC7698381; doi:10.3390/cancers12113403)
Supplement: Supplementary file 1 [file cancers-12-03403-s001.pdf]

Integrated Multi-Tumor Radio-Genomic Marker of Outcomes in Patients with High Serous Ovarian Carcinoma

Harini Veeraraghavan, Herbert Alberto Vargas, Alejandro-Jimenez Sanchez, Maura Micco, Eralda Mema, Yulia Lakhman, Mireia Crispin-Ortuzar, Erich P. Huang, Douglas A. Levine, Rachel N. Grisham, Nadeem Abu-Rustum, Joseph O. Deasy, Alexandra Snyder, Martin L. Miller, James D. Brenton and Evis Sala

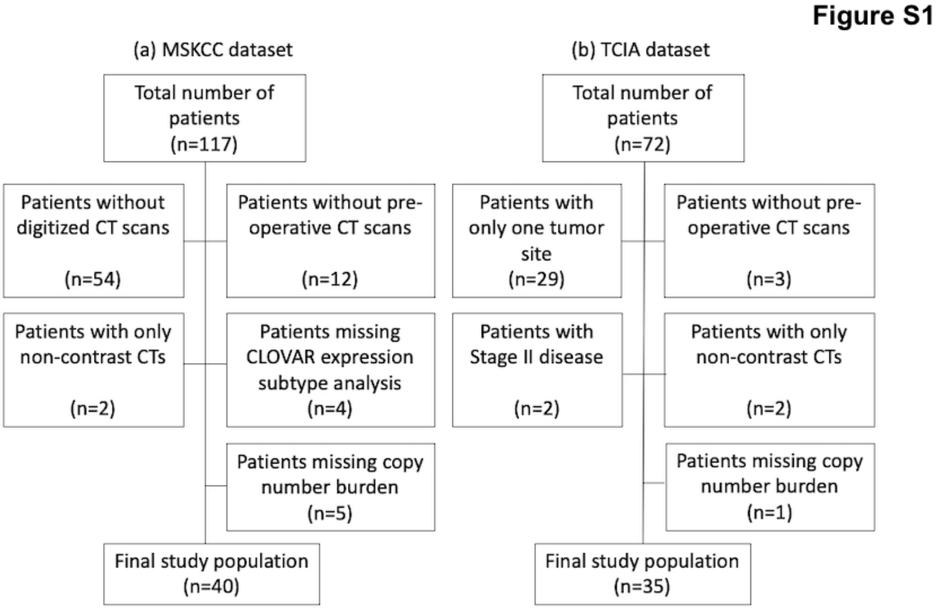

Figure S1. REMARK diagram showing patient selection.

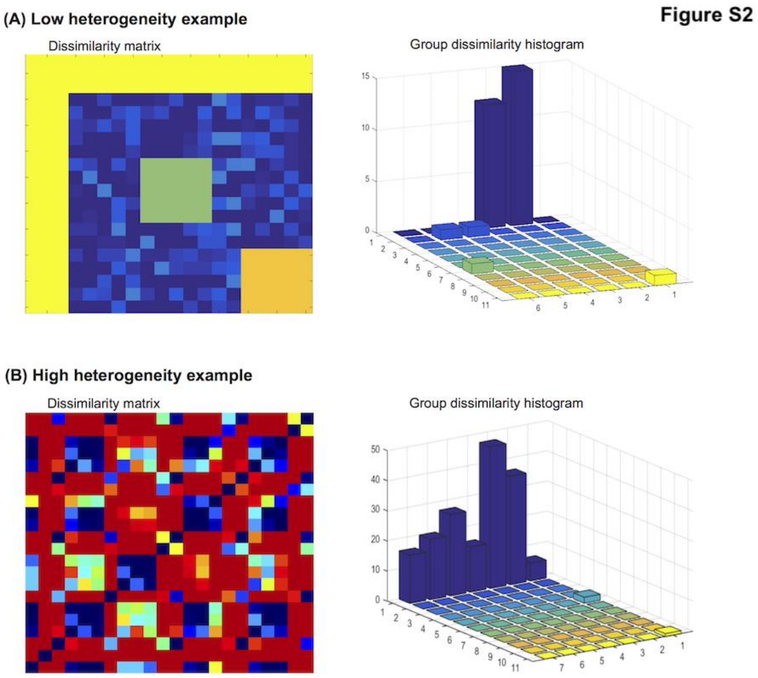

Figure S2. Differences between patients with (A) low intra- and inter-site tumor heterogeneity captured using the dissimilarity matrix and group dissimilarity matrix and (B) high intra-, and inter-site tumor heterogeneity captured using the dissimilarity matrix and group dissimilarity matrix histogram for computing the IITH measures.

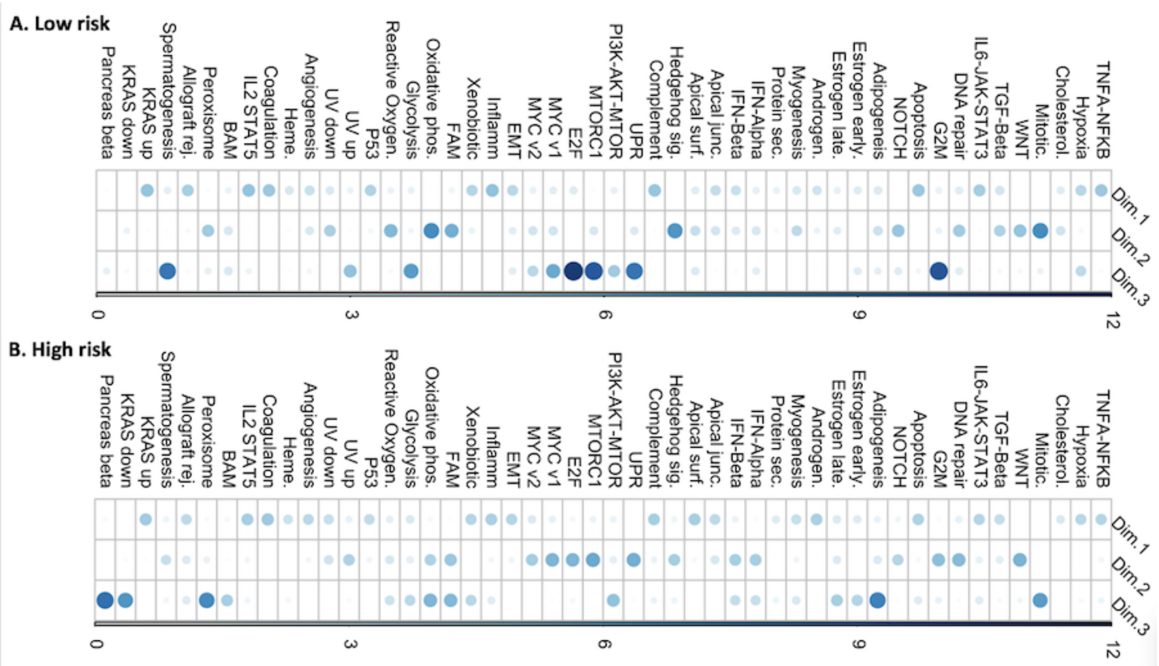

Figure S3. Principal component analysis (PCA) factor loadings of the 50 Hallmark gene sets along the first three dimensions (70% variation explained) in the (A) low-risk (cluDiss < median (68.6)) and (B) high-risk (cluDiss ≥ median) groups.

Table S1. Robustness of features to scanner differences. Wilcoxon rank-sum tests performed between GE vs. non-GE scanners. Robust features are indicated in bold font.

Feature	*p*-value	GE Median (IQR)	Non-GE Median (IQR)	Feature Category
CluDiss	0.06	70.0 (45.2, 90.3)	53.8 (37.3, 66.4)	IISTH
CT Mean	0.03	76.1 (59.1, 1053.0)	1057.0 (1040.0, 1072.0)	First order
CT SD	0.001	23.2 (19.6, 29.4)	29.1 (27.4, 39.0)	First order
CT Skewness	0.035	−0.203 (−0.41, 0.029)	−0.59 (−1.36, −0.32)	First order
CT Kurtosis	0.011	0.62 (0.17, 1.91)	2.61 (0.88, 19.6)	First order
Energy	0.001	0.011 (0.01, 0.02)	0.03 (0.02, 0.16)	GLCM
Entropy	0.003	6.95 (6.20, 7.54)	5.67 (3.30, 6.52)	GLCM
Contrast	0.002	10.7 (5.07, 16.3)	3.27 (0.81, 7.60)	GLCM
Homogeneity	0.001	0.33 (0.27, 0.42)	0.52 (0.41, 0.75)	GLCM
Correlation	0.004	0.39 (0.31, 0.49)	0.55 (0.45, 0.58)	GLCM
Short run emphasis (SRE)	0.002	0.92 (0.88, 0.94)	0.83 (0.69, 0.89)	GLRLM
Long run emphasis (LR)	0.001	1.42 (1.30, 1.68)	2.17 (1.63, 6.33)	GLRLM
Gray level non-uniformity (GLN)	0.007	39664 (15913, 105653)	140416 (82103, 545518)	GLRLM
Run length non-uniformity (RLN)	0.049	314665 (165,745, 559,879)	676622 (407,981, 868,718)	GLRLM
Run percentage (RP)	0.002	11.6 (10.9, 11.9)	10.0 (7.12, 11.0)	GLRLM
Low gray run-length emphasis (LGLRE)	0.066	0.004 (0.003, 0.006)	0.0025 (0.002, 0.005)	GLRLM
High gray run-length emphasis (HGLRE)	0.15	331.0 (258.0, 430.0)	430.0 (289.0, 536.0)	GLRLM
Short run low gray level emphasis (SRLGLE)	0.022	0.004 (0.002, 0.006)	0.0025 (0.002, 0.003)	GLRLM
Short run high gray level emphasis (SRHGLE)	0.378	313.0 (244.0, 373.0)	340.0 (251.0, 438.0)	GLRLM

Long run low gray level emphasis (LRLGLE)	0.354	0.005 (0.004, 0.008)	0.007 (0.004, 0.009)	GLRLM
Long run high gray level emphasis (LRHGLE)	<0.001	431.0 (321.0, 645.0)	1110.0 (618.0, 2094.0)	GLRLM
Gray level variance (GLV)	0.017	9.32 (6.22, 13.30)	4.23 (1.33, 9.08)	GLRLM
Run length variance (RLV)	0.001	0.15 (0.10, 0.26)	0.49 (0.25, 2.89)	GLRLM
Small area emphasis (SAE)	<0.001	0.64 (0.59, 0.65)	0.53 (0.49, 0.61)	GLSZM
Large area emphasis (LAE)	0.003	5203 (217.0, 38750.0)	188438.0 (21937.0, 2232144)	GLSZM
Gray level non-uniformity (GLN)	0.25	483.0 (238.0, 784.0)	812.0 (362.0, 1022.0)	GLSZM
Size zone non-uniformity (SZN)	0.26	2048.0 (1119.0, 3560.0)	1243.0 (851.0, 2578.0)	GLSZM
Zone prominence (ZP)	0.002	0.20 (0.11, 0.31)	0.06 (0.02, 0.14)	GLSZM
Low gray level zone emphasis (LGLZE)	0.09	0.005 (0.003, 0.007)	0.004 (0.002, 0.006)	GLSZM
High gray level zone emphasis (HGLZE)	0.26	329.0 (267.0, 427.0)	383.0 (284.0, 523.0)	GLSZM
Small zone low gray level zone emphasis (SZLGLE)	0.036	0.003 (0.002, 0.005)	0.003 (0.001, 0.003)	GLSZM
Small zone high gray level zone emphasis (SZHGLE)	0.94	218.0 (175.0, 259.0)	206.0 (157.0, 274.0)	GLSZM
Large zone low gray level zone emphasis (LZLGLE)	0.006	17.6 (0.63, 182.0)	415.0 (42.1, 7273.0)	GLSZM
Large zone high gray level zone emphasis (LZHGLE)	0.001	1955751 (63,192, 9,416,443)	95504884 (10109217,1.04e9)	GLSZM
Gray level variance (GLV)	0.023	18.5 (13.7, 22.1)	12.6 (7.05, 15.5)	GLSZM
Size zone variance (SZV)	0.003	5180.0 (207.0, 38,708.0)	188,182.0 (21,882.0, 2,225,096)	GLSZM
Contrast	0.007	0.038 (0.019, 0.069)	0.01 (0.001, 0.03)	NGTDM
Coarseness	0.519	0.00 (0.00, 0.00)	0.00 (0.00, 0.00)	NGTDM
Busyness	0.045	6.8 (3.37, 18.7)	18.2 (10.2, 27.6)	NGTDM
Complexity	0.003	900.0 (638.0, 1082.0)	539.0 (369.0, 763.0)	NGTDM
Strength	0.164	0.094 (0.06, 0.19)	0.06 (0.04, 0.11)	NGTDM
Low dependence emphasis (LDE)	0.004	0.048 (0.03, 0.08)	0.018 (0.007, 0.04)	NGLDM
High dependence emphasis (HDE)	0.002	195.0 (108.0, 353.0)	701.0 (295.0, 2711.0)	NGLDM
Low gray level count emphasis (LGCE)	0.073	0.004 (0.003, 0.006)	0.0025 (0.002, 0.005)	NGLDM
High gray level count emphasis (HGCE)	0.136	332.0 (257.0, 431.0)	433.0 (286.0, 542.0)	NGLDM
Low dependence low gray level emphasis (LDLGE)	0.069	0.0 (0.0, 0.001)	0.0 (0.0, 0.0)	NGLDM
Low dependence high gray level emphasis (LDHGE)	0.013	17.1 (9.7, 26.0)	8.12 (3.74, 14.3)	NGLDM
High dependence low gray level emphasis (HGLGE)	0.019	0.56 (0.36, 1.26)	1.76 (0.62, 3.72)	NGLDM
High dependence high gray level emphasis (HGHGE)	< 0.001	54603 (33050.0, 117209.0)	276225 (131160.0, 935277.0)	NGLDM
Gray level non-uniformity (GLN)	0.006	3467.0 (1342.0, 10546.0)	20071.0 (7847.0, 82499.0)	NGLDM
Dependence count non-uniformity (DCN)	0.107	1359.0 (799.0, 2513.0)	2703.0 (1411.0, 3722.0)	NGLDM
Grey level variance (GLV)	0.013	8.98 (5.88, 12.9)	4.02 (0.97, 8.44)	NGLDM
Dependence count variance (DCV)	< 0.001	44.9 (27.9, 86.6)	182.0 (83.7, 528.0)	NGLDM
Dependence count energy (DCEnergy)	0.027	0.005 (0.005, 0.006)	0.0045 (0.004, 0.006)	NGLDM
Dependence count entropy (DCEntropy)	0.016	7.86 (7.67, 8.04)	8.09 (7.82, 8.28)	NGLDM

Peak	0.003	166.0 (134.0, 1192.0)	1196.0 (1127.0, 1317.0)	
Valley	0.024	-1.0 (-44.0, 639.0)	674.0 (315.0, 905.0)	
Mean Sobel	0.56	99.3 (81.9, 115.0)	95.2 (85.7, 123.0)	Edge
SD Sobel	0.005	58.7 (46.8, 71.7)	70.8 (63.1, 94.0)	Edge
Skewness Sobel	0.005	1.10 (0.91, 1.58)	2.18 (1.22, 6.80)	Edge
Kurtosis Sobel	0.006	5.39 (4.27, 7.83)	14.4 (6.32, 102.0)	Edge
Mean Gabor ($0^{\circ}, \sqrt{2}$)	0.007	155.0 (146.0, 161.0)	164.0 (158.0, 169.0)	Edge
SD Gabor ($0^{\circ}, \sqrt{2}$)	0.014	3.59 (2.28, 5.77)	6.0 (4.50, 8.22)	Edge
Skewness Gabor ($0^{\circ}, \sqrt{2}$)	0.036	−0.21 (−0.58, −0.03)	-0.88 (−1.44, −0.26)	Edge
Kurtosis Gabor ($0^{\circ}, \sqrt{2}$)	0.107	3.46 (2.96, 4.66)	4.26 (3.34, 7.03)	Edge
Mean Gabor ($45^{\circ}, \sqrt{2}$)	0.011	153.0 (144.0, 160.0)	162.0 (157.0, 165.0)	Edge
SD Gabor ($45^{\circ}, \sqrt{2}$)	<0.001	2.77 (1.73, 4.92)	6.97 (5.37, 9.11)	Edge
Skewness Gabor ($45^{\circ}, \sqrt{2}$)	0.45	−0.28 (−0.81, −0.01)	−0.51 (−0.98, −0.18)	Edge
Kurtosis Gabor ($45^{\circ}, \sqrt{2}$)	0.45	4.01 (3.10, 6.62)	3.59 (2.85, 4.70)	Edge
Mean Gabor ($90^{\circ}, \sqrt{2}$)	0.25	151.0 (144.0, 159.0)	157.0 (151.0, 159.0)	Edge
SD Gabor ($90^{\circ}, \sqrt{2}$)	<0.001	2.18 (1.49, 5.18)	7.66 (6.28, 8.61)	Edge
Skewness Gabor ($90^{\circ}, \sqrt{2}$)	0.85	−0.23 (−1.04, 0.02)	−0.64 (−0.93, 0.07)	Edge
Kurtosis Gabor ($90^{\circ}, \sqrt{2}$)	0.81	4.18 (3.21, 6.60)	3.81 (3.26, 5.53)	Edge
Mean Gabor ($135^{\circ}, \sqrt{2}$)	0.031	141.0 (126.0, 148.0)	148.0 (140.0, 157.0)	Edge
SD Gabor ($135^{\circ}, \sqrt{2}$)	< 0.001	5.73 (1.83, 8.28)	12.20 (9.93, 13.60)	Edge
Skewness Gabor ($135^{\circ}, \sqrt{2}$)	0.125	−0.065 (−0.37, 0.35)	−0.24 (-0.57, −0.14)	Edge
Kurtosis Gabor ($135^{\circ}, \sqrt{2}$)	0.18	3.46 (2.50, 5.70)	2.71 (2.40, 3.57)	Edge

Table footer:

Table S2. Spearman correlation of identified robust radiomics features with TTV and number of sites.

Feature	Spearman Correlation TTV	Spearman Correlation Number of Sites	Feature Category
CluDiss	-0.040	0.833	IISTH
Low gray run-length emphasis (LGLRE)	0.019	-0.193	GLRLM
High gray run-length emphasis (HGLRE)	0.277	0.107	GLRLM
Short run high gray level emphasis (SRHGLE)	0.062	0.193	GLRLM
Long run low gray level emphasis (LRLGLE)	0.152	−0.192	GLRLM
Gray level non-uniformity (GLN)	0.717	−0.051	GLSZM
Size zone non-uniformity (SZN)	0.190	0.118	GLSZM
Low gray level zone emphasis (LGLZE)	−0.009	−0.184	GLSZM
High gray level zone emphasis (HGLZE)	0.284	0.106	GLSZM
Small zone high gray level zone emphasis (SZHGLE)	0.266	0.168	GLSZM
Coarseness	−0.245	−0.005	NGTDM
Strength	−0.303	−0.007	NGTDM
Low gray level count emphasis (LGCE)	0.014	−0.195	NGLDM
High gray level count emphasis (HGCE)	0.274	0.107	NGLDM
Low dependence low gray level emphasis (LDLGE)	-0.312	0.003	NGLDM
Dependence count non-uniformity (DCN)	0.606	−0.039	NGLDM
Mean Sobel	−0.008	−0.392	Edge

Kurtosis Gabor (0°, $\sqrt{2}$)	0.240	−0.173	Edge
Skewness Gabor (45°, $\sqrt{2}$)	0.097	−0.188	Edge
Kurtosis Gabor (45°, $\sqrt{2}$)	0.227	−0.105	Edge
Mean Gabor (90°, $\sqrt{2}$)	−0.23	0.262	Edge
Skewness Gabor (90°, $\sqrt{2}$)	0.155	−0.036	Edge
Kurtosis Gabor (90°, $\sqrt{2}$)	0.207	−0.127	Edge
Skewness Gabor (135°, $\sqrt{2}$)	0.135	0.018	Edge
Kurtosis Gabor (135°, $\sqrt{2}$)	0.181	−0.266	Edge

Table S3. Correlation of CluDiss and relevant (to platinum resistance classification) average radiomic measures to Hallmark gene sets. Correlations > abs (0.30) with significant ($p < 0.05$) correlations are considered significant.

Hallmark Gene Set	Spearman Correlation					Process Category
	cluDiss			DCN. NGTDM	GLN. GLSZM	
	All Sites	Abdomen	Pelvis			
Allograft rejection	0.187	0.354 (0.018)	−0.082	−0.356 (0.02)	−0.345 (0.022)	Immune
Complement	0.136	0.293	−0.072	−0.349 (0.02)	−0.353 (0.018)	Immune
Interferon Alpha response	0.114	0.221	0.014	−0.458 (0.002)	−0.421 (0.004)	Immune
Interferon Gamma response	0.159	0.296	−0.030	−0.496 (0.0007)	−0.45 (0.002)	Immune
IL6 JAK STAT3 signaling	0.116	0.265	−0.070	−0.335 (0.03)	−0.305 (0.044)	Immune
Inflammatory response	0.181	0.299	−0.014	−0.302 (0.05)	−0.299	Immune
TNFA Signaling via NFKB	0.067	0.191	0.035	−0.243	−0.266	Immune
IL2 STAT5 signaling	0.079	0.228	−0.093	−0.332 (0.03)	−0.355 (0.018)	Immune
WNT Beta Catenin signaling	−0.35 (0.02)	−0.399 (0.007)	−0.252	0.111	0.045	Oncogenic
MTORC1 signaling	0.29	0.377 (0.012)	0.137	−0.151	−0.143	Oncogenic
NOTCH signaling	−0.29	−0.44 (0.003)	−0.120	0.134	0.079	Oncogenic
PI3K AKT MTOR signaling	0.15	0.107	0.001	−0.305 (0.04)	−0.245	Oncogenic
Glycolysis	0.255	0.295	0.053	−0.109	−0.126	Oncogenic
E2F targets	0.093	0.088	0.033	0.077	0.142	Oncogenic
G2M checkpoint	0.079	0.036	0.037	0.068	0.128	Oncogenic
MYC targets v1	0.105	0.119	0.071	0.013	0.048	Oncogenic
MYC targets v2	0.174	0.105	0.217	−0.029	0.007	Oncogenic
P53 pathway	0.052	0.218	−0.028	−0.209	−0.247	Oncogenic
Mitotic spindle	−0.097	−0.140	−0.011	0.00028	0.038	Oncogenic
KRAS signaling up	0.052	0.213	−0.144	−0.23	−0.264	Oncogenic
KRAS signaling down	−0.16	−0.286	−0.008	0.069	0.060	Oncogenic
HEDGEHOG signaling	−0.27	−0.269	−0.226	0.091	0.063	Oncogenic
DNA repair	0.126	0.024	0.051	0.019	0.071	Oncogenic
Apical junction	0.013	0.028	−0.102	−0.016	−0.212	Stromal
Apical surface	0.027	0.075	−0.172	−0.061	−0.081	Stromal
Angiogenesis	0.086	0.236	−0.068	−0.258	−0.304 (0.044)	Stromal
Epithelial mesenchymal transition	0.099	0.214	−0.115	−0.022	−0.256	Stromal
Hypoxia	0.018	0.167	−0.125	−0.115	−0.16	Cellular

Unfolded protein response	0.058	0.139	−0.045	0.11	0.138	Cellular
Reactive oxygen species pathway	0.255	0.411 (0.006)	0.112	−0.265	−0.268	Cellular
TGF Beta signaling	−0.087	−0.047	−0.147	−0.099	−0.162	Cellular
UV response down	−0.149	−0.057	−0.176	−0.071	0.141	Cellular
UV response up	0.107	0.254	0.134	−0.107	−0.098	Cellular
Peroxisome	0.341 (0.02)	0.345 (0.022)	0.252	0.006	−0.014	Other
Fatty acid metabolism	0.36 (0.02)	0.555 (<0.0001)	0.172	−0.135	−0.157	Other
Adipogenesis	0.162	0.331 (0.028)	0.028	−0.272	−0.343 (0.023)	Other
Cholesterol homeostasis	0.153	0.329 (0.029)	0.154	−0.266	−0.308 (0.042)	Other
Xenobiotic metabolism	0.164	0.359 (0.016)	0.024	−0.202	−0.229	Other
Myogenesis	−0.075	−0.016	−0.126	−0.137	−0.201	Other
Pancreas Beta cells	−0.048	0.072	−0.257	−0.028	−0.116	Other
Coagulation	0.108	0.281	−0.071	−0.323 (0.03)	−0.338 (0.025)	Other
Bile acid metabolism	0.03	0.158	−0.025	−0.101	−0.159	Other
Heme metabolism	0.163	0.196	0.023	−0.127	−0.161	Other
Oxidative phosphorylation	0.23	0.241	0.180	−0.077	−0.082	Other
Apoptosis	0.139	0.249	−0.080	−0.289	−0.299	Other
Protein secretion	0.141	0.153	−0.209	0.098	0.089	Other
Androgen response	0.028	0.085	−0.117	−0.131	−0.222	Other
Estrogen response early	0.015	−0.038	0.075	−0.081	−0.153	Other
Estrogen response late	0.015	0.056	0.026	−0.164	−0.203	Other
Spermatogenesis	0.016	−0.073	0.039	0.033	0.090	Other

Table footer:.

Table S4. Correlation of CluDiss and relevant (to platinum resistance classification) average radiomic measures to consensus TME cell types. Correlations > abs(0.30) with significant (P < 0.05) correlations are considered significant.

Consensus TME.	Spearman Correlation				
	cluDiss			DCN.	GLN.
	All Sites	Abdomen	Pelvis	NGTDM	GLSZM
T_{gd}	0.213	0.402 (0.007)	−0.048	−0.456 (0.002)	−0.419 (0.005)
T_{regs}	0.207	0.391 (0.009)	−0.089	−0.416 (0.005)	−0.385 (0.009)
Bcells	0.202	0.395 (0.008)	−0.077	−0.407 (0.006)	−0.372 (0.012)
CD4	0.234	0.408 (0.006)	−0.105	−0.412 (0.005)	−0.389 (0.009)
CD8	0.209	0.399 (0.007)	−0.097	−0.420 (0.004)	−0.401 (0.007)
Cytotoxic	0.262	0.437 (0.003)	0.022	−0.344 (0.022)	−0.319 (0.035)
Dendritics	0.153	0.323 (0.033)	−0.113	−0.327 (0.030)	−0.299
Endothelial	0.009	0.236	−0.225	−0.228	−0.219
Eosinophils	0.173	0.286	−0.101	−0.316 (0.037)	−0.299
Fibroblast	0.046	0.223	−0.131	−0.206	−0.219
M0	0.117	0.278	−0.137	−0.273	−0.254
M1	0.180	0.331 (0.028)	−0.099	−0.339 (0.024)	−0.310 (0.040)
M2	0.171	0.328 (0.029)	−0.101	−0.320 (0.034)	−0.299
Mast	0.141	0.333 (0.027)	−0.041	−0.253	−0.238
Monocytes	0.119	0.282	−0.127	−0.278	−0.265
Neutrophils	0.173	0.338 (0.025)	−0.064	−0.297	−0.285
NK	0.254	0.426 (0.004)	−0.038	−0.445 (0.002)	−0.407 (0.006)
Plasma	0.248	0.424 (0.004)	−0.136	−0.256	−0.242

© 2020 by the authors. Licensee MDPI, Basel, Switzerland. This article is an open access article distributed under the terms and conditions of the Creative Commons Attribution (CC BY) license (http://creativecommons.org/licenses/by/4.0/).
